# Trastuzumab-based palliative chemotherapy for HER2-positive gastric cancer: a single-center real-world data

**DOI:** 10.1186/s12885-021-08058-2

**Published:** 2021-03-26

**Authors:** Tae-Hwan Kim, Hun Do Cho, Yong Won Choi, Hyun Woo Lee, Seok Yun Kang, Geum Sook Jeong, Jin-Hyuk Choi, Mi Sun Ahn, Seung-Soo Sheen

**Affiliations:** 1 Department of Hematology-Oncology, Ajou University School of Medicine, 164, World cup-ro, Yeongtong-gu, Suwon, 443-380 South Korea; 2 Department of Pulmonology and Critical Care Medicine, Ajou University School of Medicine, 164, World cup-ro, Yeongtong-gu, Suwon, 443-380 South Korea

**Keywords:** Trastuzumab, Chemotherapy, Gastric cancer, Prognosis

## Abstract

**Background:**

Since the results of the ToGA trial were published, trastuzumab-based chemotherapy has been used as the standard first-line treatment for HER2-positive recurrent or primary metastatic gastric cancer (RPMGC). However, the real-world data has been rarely reported. Therefore, we investigated the outcomes of trastuzumab-based chemotherapy in a single center.

**Methods:**

This study analyzed the real-world data of 47 patients with HER2-positive RPMGC treated with trastuzumab-based chemotherapy in a single institution.

**Results:**

With the median follow-up duration of 18.8 months in survivors, the median overall survival (OS) and progression-free survival were 12.8 and 6.9 months, respectively, and the overall response rate was 64%. Eastern Cooperative Oncology Group performance status 2 and massive amount of ascites were independent poor prognostic factors for OS, while surgical resection before or after chemotherapy was associated with favorable OS, in multivariate analysis. In addition, 5 patients who underwent conversion surgery after chemotherapy demonstrated an encouraging median OS of 30.8 months, all with R0 resection.

**Conclusions:**

Trastuzumab-based chemotherapy in patients with HER2-positive RPMGC in the real world demonstrated outcomes almost comparable to those of the ToGA trial. Moreover, conversion surgery can be actively considered in fit patients with a favorable response after trastuzumab-based chemotherapy.

## Background

Gastric cancer (GC) is the most commonly newly diagnosed type of cancer in Korea and the 2nd leading cause of cancer-related mortality in the world [1, 2]. The outcome of recurrent or primary metastatic GC (RPMGC) is poor; the median overall survival (OS) is approximately 4 months with best supportive care and approximately 11–12 months with palliative chemotherapy [3, 4]. Combination chemotherapy with a two or three-drug regimen, including platinum, fluoropyrimidine, irinotecan, or taxane has been the mainstay in the treatment of RPMGC [5–7].

While trastuzumab has been used in the treatment of human epidermal growth factor receptor 2 (HER2)-positive breast cancer, several studies have focused on its effectiveness on patients with HER2-positive GC due to the 8.2–15.6% positivity of HER2 expression status [8–10]. Since the benefit of adding trastuzumab to standard chemotherapy in metastatic HER2-positive GC was demonstrated in the ToGA trial, trastuzumab-based chemotherapy has been used as the standard first-line treatment in patients with HER2-positive RPMGC for approximately 10 years [11]. However, the real-world data concerning trastuzumab-based palliative chemotherapy for RPMGC has been rarely reported.

Therefore, we investigated the outcomes of patients with RPMGC treated with trastuzumab-based chemotherapy in a single center, while analyzing the potential prognostic factors.

## Methods

### Patients

All histologically documented advanced HER2-positive gastric adenocarcinoma patients who had initiated first-line palliative trastuzumab-based chemotherapy between June 2011 and December 2019 at Ajou University Hospital, Suwon, Korea, were retrospectively identified. The eligible patients were those with recurrent disease or American Joint Committee on Cancer (AJCC) stage IV [12] with distant metastasis, Eastern Cooperative Oncology Group (ECOG) performance status (PS) 0–2, and sufficient hepatorenal function. Eligible HER2-positive patients had the result of HER2 immunohistochemistry (IHC) 3+ or silver-enhanced in situ hybridization (SISH) positive with IHC 2+. HER2 testing and interpretation had been performed at pathology laboratory of our institution by consistent criteria, using Ventana Benchmark XT system (Ventana Medical Systems, Inc. Tucson, Arizona, USA), according to gastroesophageal adenocarcinoma guideline from the College of American Pathologists, American Society for Clinical Pathology, and American Society of Clinical Oncology [13].

Doses and intervals between courses of chemotherapy were the same as reported in the ToGA trial, with some modifications according to the discretion of physicians in cases of chemotherapy-induced toxicity [11]. The responses to chemotherapy were evaluated every 2 or 3 cycles (6 or 9 weeks) with abdominal computed tomography (CT) with or without chest CT according to the discretion of medical oncologists.

This research protocol was approved by the institutional review board (IRB) of Ajou University Hospital (IRB approval no. AJIRB-MED-MDB-20). Informed consent was waived by the IRB because this study was conducted using medical records of anonymized patients. Some patients (*n* = 9) in this study cohort were included in previous studies about palliative chemotherapy for RPMGC [4, 14, 15]. However, the follow-up of the patients was extended in the current study.

### Clinical review

We retrospectively reviewed the medical records of the eligible patients and collected data such as age, gender, ECOG PS, disease status (primary or recurrent), histologic type, HER2 status (IHC 3+ or IHC 2+ with SISH +), liver metastasis, peritoneal metastasis, amount of ascites, types of chemotherapy combined with trastuzumab, number of administration of each chemotherapeutic agent, trastuzumab maintenance status, use of surgical resection, overall response, progression status, and survival information.

The amount of ascites was classified into four categories according to previous reports: no (not observed in the CT scan), small (limited to the pelvic cavity or around the liver), moderate (definitely present on CT scan, but, neither small nor large), massive (continuous from the liver surface to the pelvic cavity) [16, 17].

Trastuzumab maintenance was defined as the continuation of trastuzumab after completing six or more cycles of triplet chemotherapy (capecitabine or 5-FU/cisplatin/trastuzumab) without evidence of disease progression, including treatment with capecitabine, in addition to trastuzumab. Conversion surgery was defined as surgical resection performed after palliative chemotherapy when complete resection, including metastatic lesions, appeared possible according to the discretion of surgeons [18].

As a historical control cohort, we investigated the outcome of 164 RPMGC patients who initiated palliative chemotherapy with non-trastuzumab containing regimen between June 2011 and December 2014. These patients were included in the cohort of previous published study [4].

### Statistical analysis

OS and progression-free survival (PFS) were calculated using the Kaplan–Meier method. The log rank test was used to analyze the differences between the survival curves. PFS was defined as the time from the starting day of trastuzumab-based chemotherapy to disease progression or the death of any cause. OS was defined as the time from the starting day of chemotherapy to death, while data on the survivors were censored at the last follow-up. Cox proportional hazards regression model was used to determine the factors influencing PFS and OS, and factors with *p* values < 0.1 in the univariate analysis were included. All statistical analyses were two-sided and performed using IBM SPSS Statics software (version 23). In addition, responses to chemotherapy were evaluated by response evaluation criteria in solid tumors version 1.1 [19].

## Results

### Patient characteristics

Overall, 47 patients with RPMGC received first-line palliative trastuzumab-based chemotherapy at our institution. The characteristics of patients at the initiation of trastuzumab-based chemotherapy are summarized in Table 1. Ten patients (21%) were ≥ 70 years, with a median age of 59 (36–83) years; 38 patients were male (81%); and 7 patients (15%) were in ECOG PS 2. The number of patients with primary metastatic gastric cancer was 36 (77%), while all of them were in AJCC stage IV. Furthermore, the numbers of patients with liver metastasis and peritoneal metastasis were 24 (51%) and 15(32%), respectively. In addition, the patients with no, small or moderate, and massive ascites on CT scan were 26 (55%), 16 (34%), and 5 (11%), respectively. Three patients received palliative surgical resection (gastrectomy, 2; resection of the metastatic liver lesion, 1) before chemotherapy. Surgical resection was performed in 6 patients (13%) after chemotherapy. Among them, 5 patients underwent conversion surgery after achieving partial response to chemotherapy, all with R0 resection, while 1 patient received resection of newly developed ovarian metastasis despite the response of other lesions. Re-evaluation of HER2 status following treatment was performed in surgical specimens of 5 IHC 3+ patients (conversion surgery: 4, palliative surgery: 1) who underwent surgery after chemotherapy, with still IHC 3+ in 4 patients and 2+ in 1 patient, respectively.

**Table 1:** Patient characteristics

Clinical characteristics	Total (***n*** = 47)
Age, years	
< 70	37 (79%)
≥ 70	10 (21%)
Gender	
Male	38 (81%)
Female	9 (19%)
ECOG PS	
0	12 (25%)
1	28 (60%)
2	7 (15%)
Disease status	
Primary metastatic	36 (77%)
Recurrent	11 (23%)
Histology	
Well or moderately differentiated	21 (45%)
Poorly differentiated	18 (38%)
Signet ring cell	4 (9%)
Combined, others	4 (9%)
HER2 status	
IHC 3+	40 (85%)
IHC 2+ with SISH +	7 (15%)
Liver metastasis	
No	23 (49%)
Yes	24 (51%)
Peritoneal metastasis	
No	32 (68%)
Yes	15 (32%)
Amount of ascites	
No	26 (55%)
Small or moderate	16 (34%)
Massive	5 (11%)
Surgical resection	
No	38 (81%)
Yes^a^	9 (19%)

*ECOG* Eastern Cooperative Oncology Group, *PS* performance status, *HER2* human epidermal growth factor receptor 2, *IHC* immunohistochemistry, *SISH* silver-enhanced in situ hybridization

^a^including 6 patients who underwent surgery after chemotherapy

The median number of trastuzumab administration was 8 (range, 1–56), and that of cisplatin was 6 (1–15). Thirty-five patients (74%) were given capecitabine, while 12 patients (26%) were given 5-FU, with the median number of capecitabine being 7 (range, 1–56) and 5-FU being 6 (range, 1–8). Furthermore, trastuzumab maintenance therapy was administrated to 19 patients (40%). While 15 patients received 6 cycles of triple combination chemotherapy, 10 patients underwent more than 6 cycles of triplet therapy (maximum 16 cycles) according to the discretion of medical oncologists. Second-line chemotherapy was performed in 22 patients, whereas 13 patients were treated with third or more lines of chemotherapy. In addition, no patient received other anti-HER2 agents or trastuzumab as second or more lines of treatment.

### Patient outcomes

Among 39 patients with a measurable lesion before the initiation of chemotherapy, 3 patients had a complete response and 22 patients achieved a partial response, giving the overall response rate of 64%, while 5 patients had stable disease, 5 patients had progressive disease, and 4 patients were unevaluable without follow-up imaging study due to various reasons.

**Fig. 1:**
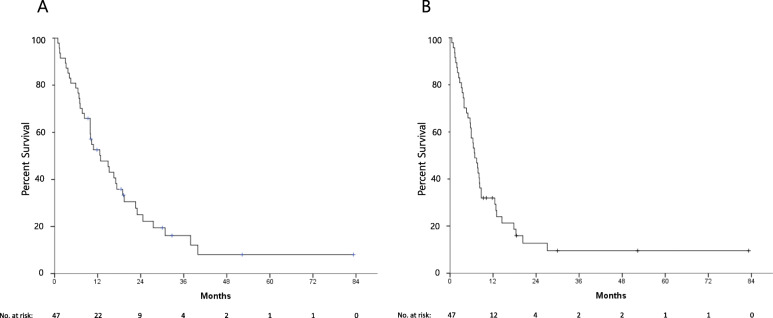
Overall survival (**a**) and progression-free survival (**b**) from the start of trastuzumab-based chemotherapy

**Fig. 2:**
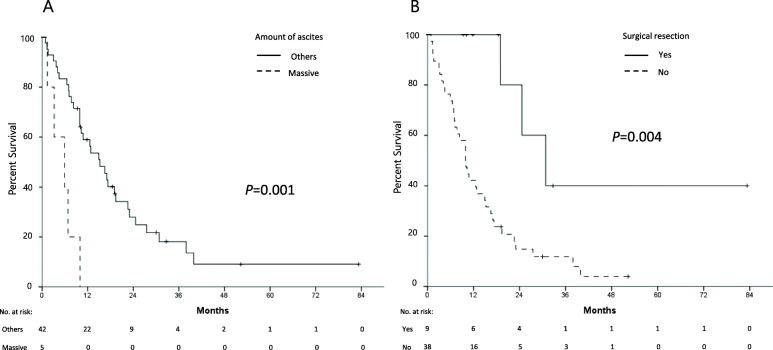
Overall survival from the start of trastuzumab-based chemotherapy according to the amount of ascites (**a**) and surgical resection (**b**)

Nine patients were alive at the last follow-up time with a median follow-up duration of 18.8 months (range, 9.3–82.9 months). The median OS and PFS were 12.8 (Fig. 1a) and 6.9 months (Fig. 1b), respectively. In the univariate analysis, the patients with ECOG PS 2 had significantly shorter median OS than those with ECOG PS 0 or 1 (*p* < 0.0001), and a massive amount of ascites also correlated with a shorter median OS (*p* = 0.001) (Fig. 2a). In addition, the patients who underwent surgical resection before or after initiation of chemotherapy showed significantly longer OS (*p* = 0.004) (Fig. 2b). ECOG PS 2 (*p* = 0.001) and massive ascites (*p* = 0.005) were independent poor prognostic factors, while surgical resection was associated with favorable OS (*p* = 0.025), in the multivariate analysis (Table 2). Univariate and multivariate analysis for PFS showed similar results (Table 2). In 5 patients with conversion surgery after chemotherapy, the median OS was 30.8 months, while 3 of them were alive at the time of analysis.

**Table 2:** Univariate and multivariate analyses of progression-free survival and overall survival

	OS					PFS				
	Univariate		Multivariate			Univariate		Multivariate		
Characteristics	Median	*p*-value	HR	95% CI	*p*-value	Median	*p*-value	HR	95% CI	*p*-value
Age, years										
< 70	15.2	0.645				7.4	0.236			
≥ 70	10.3					6.4				
Sex										
Male	12.6	0.223				7.7	0.171			
Female	14.9					3.9				
ECOG PS										
0 or 1	16.5	< 0.0001			0.001	7.7	0.007			0.028
2	4.1		5.28	2.06–13.54		2.7		2.67	1.11–6.41	
Disease status										
Primary metastatic	14.9	0.762				7.4	0.825			
Recurrent	9.9					6.4				
Histology										
Well or moderate	12.6					7.8				
Poor	12.8	0.733				5.9	0.815			
Signet ring cell	5.9					3.9				
Combined, others	4.5					3.4				
HER2 status										
IHC 3+	12.6	0.658				6.9	0.938			
IHC 2+ with SISH +	17.0					6.8				
Liver metastasis										
No	15.2	0.312				8.3	0.066			0.137
Yes	10.0					6.4		1.66	0.85–3.23	
Peritoneal metastasis										
No	14.9	0.413				6.9	0.621			
Yes	10.0					5.0				
Amount of ascites										
No/small/moderate	15.2	0.001			0.005	7.4	0.016			0.073
Massive	5.9		4.54	1.57–13.09		3.9		2.47	0.92–6.65	
Surgical resection										
No	9.9	0.004			0.025	5.9	0.002			0.022
Surgery	30.8		0.25	0.08–0.84		20.3		0.29	0.10–0.84	
Chemotherapy										
Capecitabine/Cisplatin	12.6	0.649				6.5	0.795			
5-FU/Cisplatin	12.8					8.3				

*OS* overall survival, *PFS* progression-free survival, *HR* hazard ratio, *95% CI* 95% confidence interval, *ECOG* Eastern Cooperative Oncology Group, *PS* performance status, *HER2* human epidermal growth factor receptor 2, *IHC* immunohistochemistry, *SISH* silver-enhanced in situ hybridization, *FU* fluorouracil

Cisplatin was discontinued, due to grade 3 or 4 side effects in 8 patients: acute kidney injury in 3 patients, nausea and vomiting in 2 patients, general weakness in 2 patients, and sepsis in 1 patient. The remaining drugs of the regimen, however, were continued. Four cases of treatment-related mortality occurred: pneumonia and sepsis both from neutropenia, heart failure, and cerebral infarction.

In the historical control cohort, the median OS of RPMGC patients treated with non-trastuzumab containing regimen was 11 months, while that of combination chemotherapy group (131 patients) and single agent group (33 patients) was 11 months and 9 months, respectively. In addition, almost all patients in the control cohort were HER2-negative except for 2 HER2-positive patients [4].

## Discussion

Trastuzumab-based chemotherapy has been used as a primary first-line treatment in patients with HER2-positive RPMGC at our institution since June 2011, when trastuzumab had become reimbursable from the Korean national health insurance. In this study, the median OS and PFS (12.8 and 6.9 months, respectively) of patients were almost comparable to those of the ToGA study (13.8 and 6.7 months), with similar baseline characteristics except for somewhat higher proportion of patients with ECOG 2 (15% vs. 9.8%) and 5-FU/cisplatin regimen (26% vs. 13%) [11]. In addition, the median OS (11 months) of RPMGC patients treated with non-trastuzumab combination chemotherapy in the historical control cohort was almost same as that of control group in the ToGA trial [4]. Moreover, the response to chemotherapy of this study cohort appeared higher compared to that of the ToGA trial (64% vs. 52%). Because this study was based on real-world data and the OS benefit in the Asian population of the trastuzumab-based chemotherapy in the ToGA trial was not significantly proved, these results appeared encouraging, although the number of patients was relatively small. The median OS of this study tends to be a little shorter than that of the ToGA trial, which could be attributed to the difference in the proportion of ECOG 2 patients (15% vs. 10.8%) or the relatively small number of patients in this study. However, considering the almost similar PFS between this study and the ToGA trial as well as the rather higher response rate, the findings of this study suggest that treatment in real-world settings can achieve outcomes comparable to clinical trials.

In several studies, ECOG PS 2 has been reported as a poor prognostic factor in patients with RPMGC [4, 20, 21]. In this study, the median PFS and OS of ECOG PS 2 patients were very poor (2.7 and 4.1 months, respectively), with an independent prognostic significance of ECOG PS 2 in the multivariate analysis. In addition, the benefit of adding trastuzumab was not observed in ECOG PS 2 patients in the ToGA trial [11]. Therefore, careful consideration and discussion with patients are essential before initiating trastuzumab-based chemotherapy in ECOG PS 2 patients. Considering the previous studies, including from our institution, which reported the absence of significant differences in the OS between combination and single-agent chemotherapy in ECOG PS 2 patients [4, 22–24], single-agent chemotherapy may be a useful option in ECOG PS 2 patients, even for HER2-positive cases.

A massive amount of ascites was identified as an independent poor prognostic factor for OS in this study population. Although there was no study concerning the prognostic significance of the amount of ascites in patients with RPMGC treated with trastuzumab-based chemotherapy, a poor outcome has been reported in patients with massive ascites who underwent different types of chemotherapy in some studies [17, 25]. Therefore, in patients with a massive amount of ascites, especially with ECOG PS 2, the use of trastuzumab-based chemotherapy should be carefully decided.

Surgical resection before or after chemotherapy was an independent favorable prognostic factor in this study group, although the number of patients who underwent surgery was small. The role of surgical resection in metastatic gastric cancer is controversial. The REGATTA trial, the only phase III trial in metastatic gastric cancer patients, did not demonstrate the benefit of palliative gastrectomy before chemotherapy compared to chemotherapy alone [26]. On the contrary, there were several encouraging retrospective studies for post-chemotherapy resection (i.e., conversion surgery) in patients with metastatic gastric cancer [27, 28]. Although various combination regimens were used before the conversion surgery, there were limited reports on trastuzumab-based chemotherapy [27–31]. A recent retrospective study from Japan reported favorable outcomes in 20 patients who underwent surgical resection after trastuzumab-based chemotherapy [29]. Considering the encouraging outcome of patients who received conversion surgery in this study with 30.8 months of median OS, surgical resection could be actively considered if patients show a good response after trastuzumab-based chemotherapy, amenable to complete resection.

The treatment-related mortality rate of 8% (4 patients) of this study cohort appears higher than that of the ToGA trial (3%). Given the relatively small number of patients in this real-world data, it is difficult to consider it as meaningful. However, mortality cases of infection resulting from neutropenia or heart failure suggest that careful monitoring is needed during trastuzumab-based chemotherapy. In addition, in this study, a significant number of patients (17%) experienced discontinuation of cisplatin during treatment. Replacement of cisplatin with other agents, such as oxaliplatin or irinotecan, can be considered in patients with high risk of cisplatin-induced toxicity. However, further clinical trial is necessary to establish such regimens in routine practice.

This study has some limitations. First, since it is a retrospective study with a relatively small sample size, the role of potential prognostic factors (i.e., massive amount of ascites and conversion surgery) cannot be clearly defined, requiring further larger studies. Second, the timing of the response assessment differed according to the treating medical oncologist. Third, the detailed assessment of chemotherapy-induced toxicity was not conducted as a limitation of the retrospective study. Nonetheless, the current study investigated the real-world data by analyzing the eligible patients within a certain period, while comparing with the results of the ToGA trial. Although few retrospective studies of trastuzumab-based chemotherapy have been reported, they had some limitations in reflecting the real-world data, such as studies including patients enrolled in clinical trials, treated as second-line chemotherapy, or receiving cytotoxic agent combinations not used in the ToGA trial as well as multi-center studies with a small number of patients per institution [32–36]. In addition to several clinical implications, the present study suggests that conversion surgery may achieve favorable results after trastuzumab-based chemotherapy.

## Conclusions

Trastuzumab-based chemotherapy in patients with HER2-positive RPMGC in real world demonstrated outcomes almost comparable to those of the ToGA trial. Moreover, conversion surgery can be actively considered in fit patients with a favorable response after trastuzumab-based chemotherapy.

## Data Availability

The datasets generated and/or analyzed during the current study are not publicly available due to the confidentiality of the data of patient but are available from the corresponding author on reasonable request.

## Abbreviations

HER2: Human epidermal growth factor receptor 2
GC: Gastric cancer
RPMGC: Recurrent or primary metastatic gastric cancer
OS: Overall survival
ToGA: Trastuzumab for Gastric Cancer
AJCC: American Joint Committee on Cancer
ECOG: Eastern Cooperative Oncology Group
PS: Performance status
IHC: Immunohistochemistry
SISH: Silver-enhanced in situ hybridization
CT: Computed tomography
IRB: Institutional review board
PFS: Progression-free survival
